# Supplementary material for: Deciphering the RRM-RNA recognition code: A computational analysis
Source: PLoS Comput Biol. 2023 Jan 23;19(1):e1010859. doi: 10.1371/journal.pcbi.1010859 (PMC9894542; doi:10.1371/journal.pcbi.1010859)
Supplement: S1 Fig — The two RNAs are aligned in all possible combinations using a sliding window, with the number of matches and unique positions each nucleotide interacts with in the RRM sequence counted for the 4 positions aligned in this example. The ratios between the matches and unique positions are added and then averaged for all the positions by dividing them by the length of the alignment. (PDF) [file pcbi.1010859.s001.pdf]

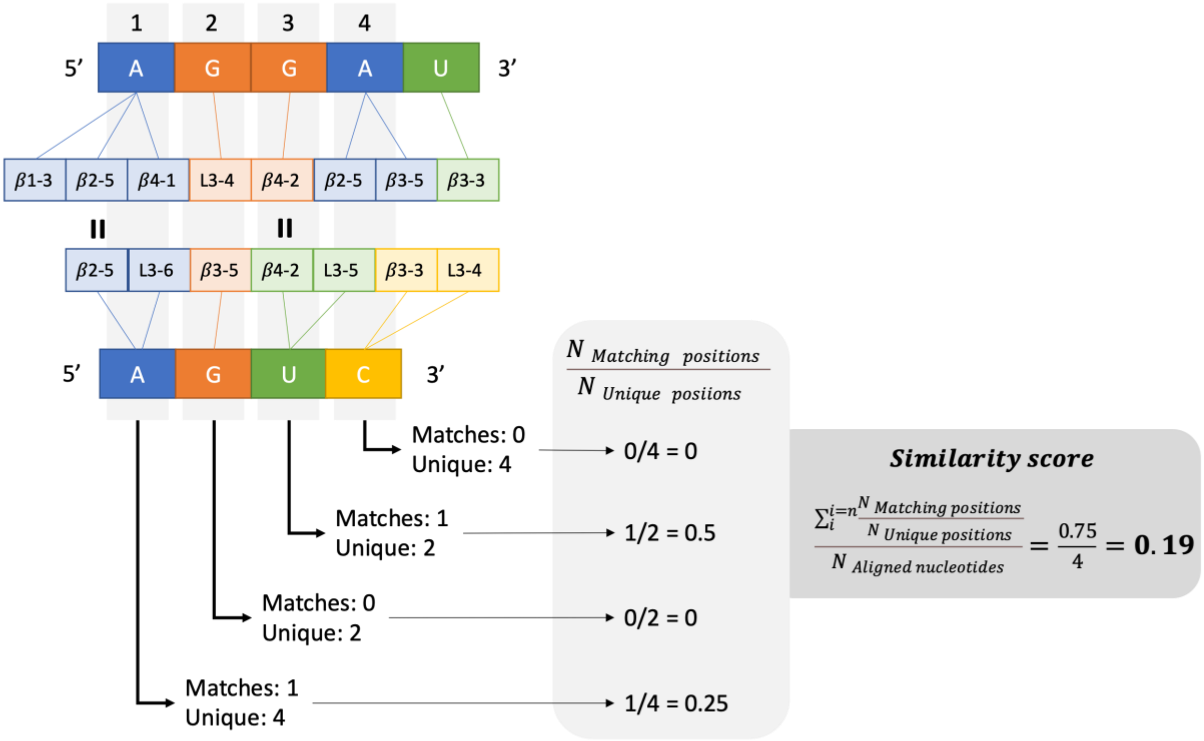

S1 Fig**:** Schematic procedure for the similarity score calculation between two RNAs based on their binding with the RRM. The two RNAs are aligned in all possible combinations using a sliding window, with the number of matches and unique positions each nucleotide interacts with in the RRM sequence counted for the 4 positions aligned in this example. The ratios between the matches and unique positions are added and then averaged for all the positions by dividing them by the length of the alignment.
