# Supplementary material for: Deciphering the RRM-RNA recognition code: A computational analysis
Source: PLoS Comput Biol. 2023 Jan 23;19(1):e1010859. doi: 10.1371/journal.pcbi.1010859 (PMC9894542; doi:10.1371/journal.pcbi.1010859)
Supplement: S2 Fig — Variation on the number of clusters (A) and number of entries in the biggest cluster (B) depending on the chosen cutoffs for similarity score (X-axis, scores from 0 to 1) and percentage of entries (Y-axis from 0% to 100%) that should have an equal or higher similarity score with the rest of the cluster. The chosen cutoff for the cluster generation is highlighted in yellow. (PDF) [file pcbi.1010859.s002.pdf]

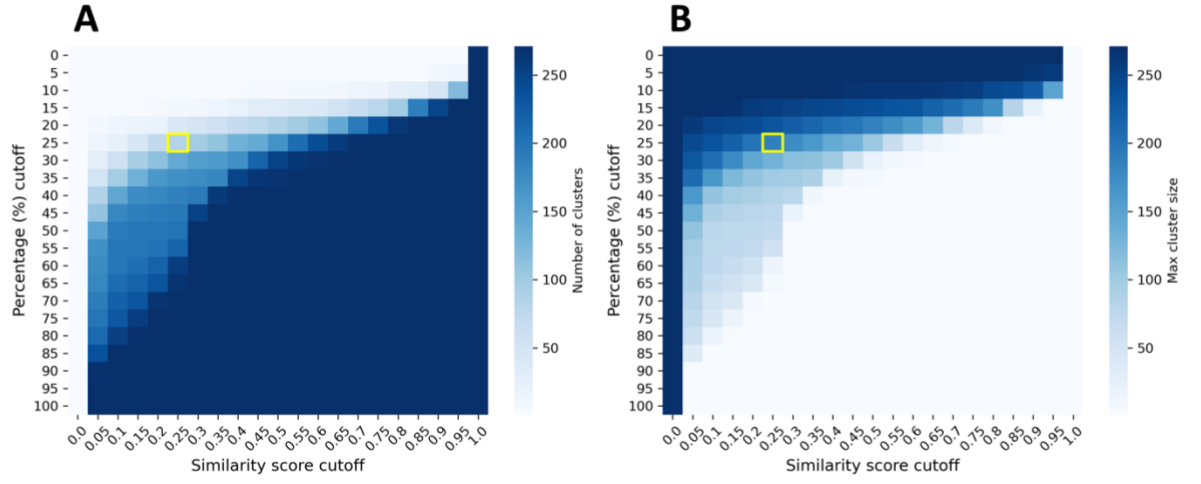

S2 Fig: Variation on the number of clusters (A) and number of entries in the biggest cluster (B) depending on the chosen cutoffs for similarity score (X-axis, scores from 0 to 1) and percentage of entries (Y-axis from 0% to 100%) that should have an equal or higher similarity score with the rest of the cluster. The chosen cutoff for the cluster generation is highlighted in yellow.
