# Supplementary material for: Deciphering the RRM-RNA recognition code: A computational analysis
Source: PLoS Comput Biol. 2023 Jan 23;19(1):e1010859. doi: 10.1371/journal.pcbi.1010859 (PMC9894542; doi:10.1371/journal.pcbi.1010859)
Supplement: S3 Fig — The aligned nucleotides used for the RMSD calculations are labelled. (PDF) [file pcbi.1010859.s003.pdf]

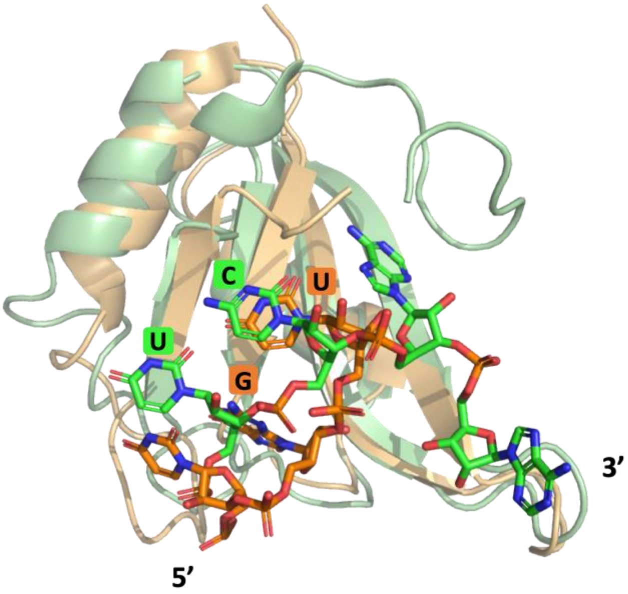

S3 Fig: Superimposed RRM-RNA complexes with the lowest similarity score in cluster 0, PDB Id. 6g90 (chain B, green) and PDB Id. 3nnh (chain B, orange). The aligned nucleotides used for the RMSD calculations are labelled.
