# Supplementary material for: Deciphering the RRM-RNA recognition code: A computational analysis
Source: PLoS Comput Biol. 2023 Jan 23;19(1):e1010859. doi: 10.1371/journal.pcbi.1010859 (PMC9894542; doi:10.1371/journal.pcbi.1010859)
Supplement: S4 Fig — (PDF) [file pcbi.1010859.s004.pdf]

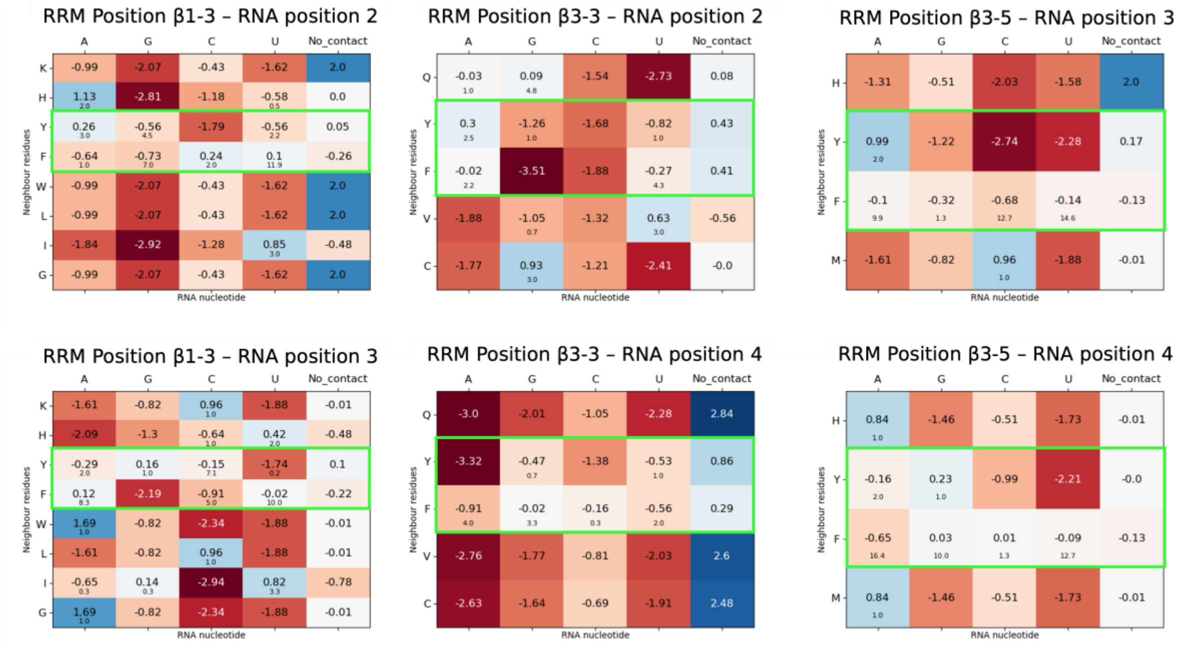

S4 Fig: Scores for the conserved aromatic positions in RNP1 (β1-3) and RNP2 (β3-3, β3-5) in contact with their respective RNA positions (phenylalanine and tyrosine scores are highlighted in bright green).
