# Supplementary material for: Deciphering the RRM-RNA recognition code: A computational analysis
Source: PLoS Comput Biol. 2023 Jan 23;19(1):e1010859. doi: 10.1371/journal.pcbi.1010859 (PMC9894542; doi:10.1371/journal.pcbi.1010859)
Supplement: S1 Table — (PDF) [file pcbi.1010859.s005.pdf]

S1 Table: PFAM identifiers and related metadata of the selected RRM families for the analysis.

PFAM ID	PFAM NAME	PFAM TYPE	PFAM CLAN ID	PFAM CLAN NAME
**PF00076**	RRM_1	Family	CL0221	RRM
**PF02994**	Transposase_22	Family	CL0221	RRM
**PF03467**	Smg4_UPF3	Family	CL0221	RRM
**PF03468**	XS	Family	CL0221	RRM
**PF03880**	DbpA	Family	CL0221	RRM
**PF04847**	Calcipressin	Family	CL0221	RRM
**PF05172**	Nup35_RRM	Family	CL0221	RRM
**PF08152**	GUCT	Family	CL0221	RRM
**PF08675**	RNA_bind	Family	CL0221	RRM
**PF08777**	RRM_3	Family	CL0221	RRM
**PF08952**	DUF1866	Family	CL0221	RRM
**PF09162**	Tap-RNA_bind	Family	CL0221	RRM
**PF11608**	Limkain-b1	Family	CL0221	RRM
**PF11835**	RRM_8	Family	CL0221	RRM
**PF13893**	RRM_5	Family	CL0221	RRM
**PF16367**	RRM_7	Family	CL0221	RRM
**PF16842**	RRM_occluded	Family	CL0221	RRM
**PF17774**	YlmH_RBD	Family	CL0221	RRM
**PF18444**	RRM_9	Family	CL0221	RRM
