# Supplementary material for: Deciphering the RRM-RNA recognition code: A computational analysis
Source: PLoS Comput Biol. 2023 Jan 23;19(1):e1010859. doi: 10.1371/journal.pcbi.1010859 (PMC9894542; doi:10.1371/journal.pcbi.1010859)
Supplement: S2 Table — (PDF) [file pcbi.1010859.s006.pdf]

S2 Table: Identifiers of the 19 selected structures to use in PROMALS3D.

UniProt Id.	PDB Id.	Chain	PFAM family
**P09012**	1FHT	A	RRM_1
**O18409**	2KHC	A	RRM_1
**F1LQ48**	2MQL	A	RRM_1
**Q9NIH4**	5ZUH	A	RRM_1
**P22626**	1X4B	A	RRM_1
**P05455**	1OWX	A	RRM_3
**Q4G0J3**	5KNW	A	RRM_3
**P26599**	2N3O	A	RRM_5
**Q8NE35**	2DNL	A	RRM_7
**Q921F4**	2E5I	A	RRM_8
**G0SET4**	4WPM	A	RRM_9
**Q99257**	4WWU	A	RRM_9
**O15056**	1UFW	A	DUF1866
**Q9JHG6**	1WEY	A	CALCIPRESSIN
**Q8R4R6**	1WWH	A	NUP35_RRM
**Q9Y4F3**	2DIU	A	MARF_RRM1
**P42305**	2G0C	A	DPA
**Q9H1J1**	2L08	A	SMG4_UPF3
**Q9NR30**	2M3D	A	GUCT
