# Supplementary material for: Deciphering the RRM-RNA recognition code: A computational analysis
Source: PLoS Comput Biol. 2023 Jan 23;19(1):e1010859. doi: 10.1371/journal.pcbi.1010859 (PMC9894542; doi:10.1371/journal.pcbi.1010859)
Supplement: S3 Table — (PDF) [file pcbi.1010859.s007.pdf]

S3 Table: Experimental Kd values correlated with the RRMScorer scores for the WT RNA and the 36 RNA mutants tested against MSI1 RRM1, using a window size of three nucleotides.

RNA SEQUENCE	SCORE	KD
UUUAUAGUUUUU	-0.300	68
AUUAUAGUUUUU	-0.300	128
CUUAUAGUUUUU	-0.300	144
GUUAUAGUUUUU	-0.300	109.3
UAUAUAGUUUUU	-0.300	142.7
UCUAUAGUUUUU	-0.300	113.7
UGUAUAGUUUUU	-0.300	96.6
UUAAUAGUUUUU	-0.300	128.7
UUCAUAGUUUUU	-0.300	95.3
UUGAUAGUUUUU	-0.300	70.9
UUUCUAGUUUUU	-0.300	92.1
UUUGUAGUUUUU	-0.300	40.3
UUUUUAGUUUUU	-0.300	66.4
UUUAAAGUUUUU	-0.518	1953.3
UUUACAGUUUUU	-0.486	1106.7
UUUAGAGUUUUU	-0.300	180.3
UUUAUCGUUUUU	-0.591	2070
UUUAUGGUUUUU	-0.481	1533.3
UUUAUUGUUUUU	-0.437	1750
UUUAUAAUUUUU	-0.518	2500
UUUAUACUUUUU	-0.514	2460
UUUAUAUUUUUU	-0.591	2223.3
UUUAUAGAUUUU	-0.300	178.7
UUUAUAGCUUUU	-0.300	235.7
UUUAUAGGUUUU	-0.300	84
UUUAUAGUAUUU	-0.300	149.3
UUUAUAGUCUUU	-0.300	133.3
UUUAUAGUGUUU	-0.300	110
UUUAUAGUUAUU	-0.300	144.3
UUUAUAGUUCUU	-0.300	131
UUUAUAGUUGUU	-0.300	102.9
UUUAUAGUUUAU	-0.300	162
UUUAUAGUUUCU	-0.300	137.7
UUUAUAGUUUGU	-0.300	91.4
UUUAUAGUUUUA	-0.300	144
UUUAUAGUUUUC	-0.300	89.6
UUUAUAGUUUUG	-0.300	99.7
