# Supplementary material for: Deciphering the RRM-RNA recognition code: A computational analysis
Source: PLoS Comput Biol. 2023 Jan 23;19(1):e1010859. doi: 10.1371/journal.pcbi.1010859 (PMC9894542; doi:10.1371/journal.pcbi.1010859)
Supplement: S1 File — Our method is based on the information difference between the occurrence of two events, in our case the information of how often a specific nucleotide interacts with a specific residue, I(Ni;RJ), and the information when that same nucleotide interacts with any other residue, I(n−Ni;RJ) (S1 Equation). The individual terms are developed in S2 and S3 Equations where: fNi,Rj is the number of occurrences for a specific contact between nucleotide i and residue j; fRj the number of times residue j is in that position; fNi the number of times nucleotide i is in that position; fn−Ni,Rj the number of times nucleotide i interacts with any residue but residue j; fn−Ni the number nucleotides other than nucleotide i in that position; R the total number of residues in the dataset. As it’s a difference between the two logarithms, the common terms that account for the number of specific residues in position j (fRj) and total number of residues in the dataset (R) disappear from the equation. After the simplification we obtain S4 Equation (Same as Eq 2 in the main text). (PDF) [file pcbi.1010859.s008.pdf]

$$I(\Delta N_i; R_J) = I(N_i; R_J) - I(n - N_i; R_J) \qquad \text{Equation S1}$$

$$I(N_i; R_J) = \ log\left(\frac{f_{N_i,R_j}/f_{R_j}}{f_{N_i}/R}\right) \qquad \text{Equation S2}$$

$$I(n - N_i; R_J) = \ log\left(\frac{f_{n-N_i,R_j}/f_{R_j}}{f_{n-N_i}/R}\right) \qquad \text{Equation S3}$$

$$I(\Delta N_i; R_J) = log\left(\frac{f_{N_i,R_j}}{f_{n-Ni,Rj}}\right) + log\left(\frac{f_{n-N_i}}{f_{N_i}}\right) \qquad \text{Equation S4}$$

S1-S4 Equations**:** Basis for the development of the RRMScorer equation. Our method is based on the information difference between the occurrence of two events, in our case the information of how often a specific nucleotide interacts with a specific residue, $I(N_i; R_J)$, and the information when that same nucleotide interacts with any other residue, $I(n - N_i; R_J)$ (equation S1). The individual terms are developed in equations S2 and S3 where: $f_{N_i,R_j}$is the number of occurrences for a specific contact between nucleotide *i* and residue *j*; $f_{R_j}$the number of times residue *j* is in that position; f_{N_i} the number of times nucleotide *i* is in that position; f_{n-N_i,R_j} the number of times nucleotide *i* interacts with any residue but residue *j*; f_{n-N_i} the number nucleotides other than nucleotide *i* in that position; R the total number of residues in the dataset. As it's a difference between the two logarithms, the common terms that account for the number of specific residues in position *j* (f_{R_j}) and total number of residues in the dataset (R) disappear from the equation. After the simplification we obtain equation S4 (Same as equation 2 in the main text).
