# Supplementary material for: Deciphering the RRM-RNA recognition code: A computational analysis
Source: PLoS Comput Biol. 2023 Jan 23;19(1):e1010859. doi: 10.1371/journal.pcbi.1010859 (PMC9894542; doi:10.1371/journal.pcbi.1010859)
Supplement: Attachment — Submitted filename: response_to_reviewers.pdf [file pcbi.1010859.s019.pdf]

Response to reviewers

We thank the reviewers for the careful reviews and constructive comments. Our answers are detailed in blue below.

Reviewer #1:

Roca-Martinez and coworkers have performed a computational analysis of RNA recognition motifs (RRMs) and RRM-RNA complexes in an effort to develop a scoring method for predicting and evaluating the probability of interaction between canonical RRMs and single-stranded RNA. The authors use available sequence and structure information to obtain individual scoring matrices for commonly observed interacting positions in RRM and RNA sequences (identified through multiple-sequence alignments), describing the preference of different nucleobase types to interact with different residue types. While the question of understanding the physicochemical underpinnings of RNA-protein interactions and predicting and sculpting their sequence determinants is extremely timely and important, the comments below should be addressed in detail before the suitability of the manuscript for publication can be adequately assessed.

Major comments:

1. The description of the RRM-RNA scoring approach (p. 6), the very heart of the manuscript, is unclear and sloppy. Equation 2 is not consistent with the text and a proper explanation of the symbols and indices used is missing (denominator in the first term different from text, fn not explained, index i in denominator in the first term different from index J in the text etc.). Also, the explanations are given in an incomplete way e.g. the sentence "…is related to the number of times adenines interact with any other amino acid residue in position beta1-1" is missing the crucial qualifier "adenines at position 1". This makes it hard to comprehend how the scores were actually calculated.

The equation is now explained in a more detailed and precise manner, and the errors in the subscripts have been fixed. The example provided has also been rephrased (page 6).

2. More importantly, the motivation and the physical foundation of the scoring function is not adequately explained. Centrally, the scores do not consider the frequency of amino-acid residues observed at a specific position (see e.g. the second term in Eq. 2), making it not symmetric when considering nucleotides and residues, respectively. In the example given on p. 6, the score should depend on the frequency of arginines interacting with adenines as well as with other nucleotides, but this is not included.

The reason why the amino acid information is not part of the equation is because the GOR method, which we adapted for this work, employs an information **difference** equation (1). This information difference between the occurrence of two events, in our case the information of how often a specific nucleotide interacts with a specific residue ($I(N_i; R_J)$) and the information when that same nucleotide interacts with any other residue ($I(n - N_i; R_J)$) is expressed by:

$$I\left(\Delta N_i; R_J\right) = I\left(N_i; R_J\right) - I\left(n - N_i; R_J\right)$$

Where the individual terms based on the information function are:

$$I\left(N_i; R_J\right) = \ log\left(\frac{f_{N_i,R_j}/f_{R_j}}{f_{N_i}/R}\right)$$

$$I\left(n - N_i; R_J\right) = \ log\left(\frac{f_{n-N_i,R_j}/f_{R_j}}{f_{n-N_i}/R}\right)$$

As we use the difference between the two logarithms, the common terms that account for the number of specific residues in position *j* (f_{R_j}) and total number of residues in the dataset (R) disappear from the equation. After the simplification we therefore obtain the following equation.

$$I\left(\Delta N_i; R_J\right) = log\left(\frac{f_{N_i,R_j}}{f_{n-Ni,Rj}}\right) + log\left(\frac{f_{n-N_i}}{f_{N_i}}\right)$$

The source of the equation is now carefully presented in the manuscript, explaining its adaptation from the GOR method in more detail. The above equations and explanatory text are also included in Supplementary Material (equations S1 to S4).

3. Also, the scoring function shares resemblance with the standard quasi-chemical approach for defining knowledge-based potentials (Miyazawa, S. and Jernigan, R.L., Macromolecules, 18, 534-552 (1985)), but with important differences. Namely, the authors here normalize the number of occurrences of a given event (e.g. presence of a nucleotide at a given site interacting with a given residue) by the number of all events other than that event (e.g the number of interactions of that nucleotide with all the other residues, except the one in question) and not the total number of all events (e.g. the number of interactions of that nucleotide with all residues). Why is this?

This is also related with the underlying principle of the information equation we are using, where the occurrence of an event is divided by the non-occurrence of that event, so in our case when the nucleotide interacts with any other residue except for the one we are calculating the information difference for.

The authors are motivated by the GOR method for analyzing secondary structural propensities, but it is not clear that the same formalism is applicable here – namely, in the GOR method one analyzes the linkage between an object (amino acid) and its property, while here one analyzes the propensity of two objects (amino acid and nucleotide) to co-occur in the same context (i.e. contact). This is related to the asymmetry discussed in point 1.

The GOR method relies on the broadly used and well described information theory principles, applying the information difference equation. Therefore, our equation is also based on those principles, but we described it as an adaptation of the GOR equation as its usefulness was

already proven for protein secondary structures in a time when few data were available. Despite the differences between the content, we think that the information difference equation is therefore applicable and useful in the RRM-RNA binding case, as illustrated in the different independent tests, and also given the limited amount of data available.

4. The rather extensive literature on contact-based statistical potentials for nucleic-acid/protein interactions should be adequately cited and discussed (see, for example Donald et al. Nucleic Acids Res., 2007, 35, 1039–1047. or Tuszynska et al. BMC Bioinformatics, 2011, 12, 348 and other).

The knowledge-based potentials are now introduced alongside the other protein/nucleic-acid prediction methods in the introduction. It is also briefly compared with our method along with reasoning why we opted for a simpler approach here.

5. The authors refer to their randomized test set as a negative test set (p. 7). As there is no guarantee that many members of this set are not actual binders – the naming should be changed to something like “background set” or “randomized set”, but certainly not “negative set”. More critically, randomization was only done on the side of the RNA sequences (change of 1 nucleotide in the sequence) and not on the side of RRM sequences – this relates to the asymmetry of the whole approach as discussed above and must be properly defended.

The negative set has been renamed to randomized set following the reviewer suggestion, as indeed, the randomly generated RNA sequence could still be a binder, a fact probably reflected by the overlap between the positive and randomized sets in Figure 6.

The sequence randomization is only applied on the RNA sequence side because our focus is on predicting which single strand RNA fragments a particular RRM might bind. Changing the protein amino acids has two key problems:

a) Our method focuses on a specific RNA binding mode, and big changes on the protein side might disrupt the RNA canonical binding.

b) Changing (many) residues on the protein can lead to a non-functional protein that might not fold as an RRM, or might not fold at all. The amino acid residues are tightly interconnected, which is not the case for the RNA: even though the RNA might form secondary structure elements that are related to its function, we are here predicting binding for exposed single strand RNAs, enabling us to rationalise the randomisation of the sequence on the RNA end.

6. Defining clusters as all complexes that have a certain similarity score with at least 25% of complexes in the cluster is quite low as a cutoff (p. 5). Of course, if one increases the cutoff, one risks not having sufficient samples for adequate statistics. The authors should defend the choice of their cutoff by providing quantitative evidence that it does not overly impact the scores i.e. the qualitative features of their method.

We have included a more detailed explanation (page 5) and plots (Figure S3) to support the chosen cutoffs. The main goal was to keep as many entries as possible in the cluster while

guaranteeing that the RNAs are still similar enough, keeping the balance between the variability and meaning of the clusters. Based on Figure S3, the selected cutoffs, 0.25 minimum similarity score and 25% similar entries in the cluster, are on the interface between i) generating many very small clusters with few entries and ii) a few big clusters with many entries that are not related. Our choice was also complemented with the RMSD check between the RNAs raising the lowest similarity score in the studied cluster (Figure S2).

7. For the validation of their scoring method the authors analyze two experimentally studied examples, while extensive data on RRM binding motifs obtained by different experimental methods exists and is not used. See for example the RNAcompete results (Ray, D., Kazan, H., Cook, K. et al, A compendium of RNA-binding motifs for decoding gene regulation, Nature, 499, 172–177 (2013)) or the Attract database (PMID: 27055826). The authors should validate their results on an as extensive a set of experimental data as possible.

We have now included a new validation section to the manuscript that uses all the RNAcompete data available for RRMs, consisting of 171 different proteins obtained from the CISBP-RNA Database. We have correlated the RNA experimental preferences with the RRMScorer predictions showing a clear correlation between both, particularly evident on single RRMs (Figure 11). The correlation for multiple RRMs and multiple RBPs is worse as there is only one RNA frequency matrix provided for each protein and in general different RRM domains (or other RBPs) contribute to the obtained nucleotide preferences. This is in fact a significant limitation when analysing biochemical binding data such as RNACompete and even more so when comparing binding data from CLIP experiments in cells where the presence of multiple domains and additional factors (including RNA binding proteins) is reflected in binding motifs identified and is often biased by the highest affinity interaction.

Minor comments

1. On p. 9, the authors state that “The unbiased number of observed contacts in the training set that is used to calculate the scores is also shown in the preference matrices (Figure 8 B,C), below each of the scores”. However, these numbers are not integers, so it is unclear what they actually refer to.

Due to the large variability in the available structures for different UniProt entries, those numbers reflect the conservation of those contacts for each UniProt ID, which are then summed. For example, an arginine-uracil contact is observed in 2 different UniProt IDs. For one of the UniProt entries there is only one available structure, so the contact contributes with 1 to the count, as this is the only contact observed. For the other UniProt entry there are 10 structures available but the contact is only observed in 8 out of the 10 structures, therefore the contribution of this UniProt ID is 0.8. The unbiased number of observed contacts for that interaction would be 1.8. With this procedure we avoid the bias towards the most studied proteins that are overrepresented in our dataset. The text has been updated to make this point clearer (page 9).

2. Numbers in Figure 2 are not fully consistent with the text (1263 instead of 1259, 20 instead of 19; p. 3 and p. 4).

The figure was updated, 1259 and 19 were indeed the right numbers for the total number of RRM domains and representative RRMs, respectively.

3. It is stated that the "alignment for the RRM-RNA structures" is available in Dataset S6 (p. 4). However, Dataset S6 contains the RRM-RNA similarity matrix.

Fixed, RRM-RNA alignment uploaded and supplementary dataset names updated.

4. In the caption of Table 1 (p. 28), the authors state that "The symbols reflect the score change after the E87N mutation", however there are no symbols.

Fixed, the missing symbols have been added.

Reviewer #2:

The paper describes construction of statistical based potential for scoring rrm domain interactions with specific RNA sequence. They use structural model of binding to identify contacting residues and then create sequence based interaction statistics. The authors acknowledge that the method is limited to already known binding modes of rna to RRM domains or very close to those. The authors validate the approach on leave out training set as well as few novel cases. The problem is still open - but there is a progress. I think the paper can be published. Would be interesting to compare the approach with AF like approach for modeling protein RNA interactions (see the link below). Also can protein rna models from this approach can be used to create alignment?

https://www.biorxiv.org/content/10.1101/2022.09.09.507333v1.full.pdf

RoseTTAFoldNA provides high accuracy models with atomic resolution for nucleic-acid complexes, which is of course extremely useful for proteins where it is clear which RNA the protein binds. However, in the case of RRMs, and probably other RBPs, it still remains a question, which RNA sequence a specific protein binds in the first place, and this is where our method can help, especially at the genome scale and for computational screening in protein design. Moreover, for many RNA binding domains, there is substantial variability in the recognition of specific nucleotides at a given position, which is captured by our analysis.

It would be possible to calculate RoseTTAFoldNA models and cluster them to create larger RRM-RNA datasets, but we prefer to use only experimental structures as ground truth for our predictor given the inevitable uncertainty of predicted models. We agree though that follow-up study to explore this would certainly be interesting. RoseTTAFoldNA is now also introduced and discussed in the manuscript.

Reviewer #3:

The manuscript by Roca-Martinez RNA-recognition motifs discusses a scoring method to estimate binding between an RRM and a single stranded RNA, and the method aims to predict RRM binding RNA sequence motifs based on RRM protein sequence. The authors adopt a simpler statistical approach over deep learning method employed in several existing methods or better interpretability. Interesting results on discriminating of high affinity RNAs with the UAG core motif from lower affinity RNAs are reported. While the reported method serves a useful purpose towards the overall task of solving the problem of deciphering RNA recognition code of RRMs, there are a number of significant issues:

1. The score described by Equation 2 is not explained and the physical underpinning cannot be found. The two log terms summed are the same as product of two ratio. But it is not clear what does it mean, and why does this make sense? Does it model some eperical binding? Conservation? Not clear.

The manuscript text has been updated to provide further details on the source and meaning of the equation (page 6) with more information available on the equation development in supplementary materials (equations S1 to S4). This point is discussed in detail in comment #2 to reviewer #1.

Furthermore, why the denominator in the first term comes to be f_n - N_i,R_J ? This is not understandable.

There was an error in the denominator in the first term as all the elements should have been in the subindex, $f_{n-Ni,Rj}$, representing any other contacts between Ni and Ri that is not the one under study. The proposed example to explain this term has also been improved to clarify it.

2. There are numerous places where the method development depends on visual inspection. This raises serious issue of reproducibility.

There are several parts in the text where visual inspection was mentioned, which we think served as a qualitative validation of the results rather than a quantitative approach on which we would base decisions - that would indeed represent a reproducibility problem. We enumerate the parts of the text where visual inspection plays a role to justify our decision:

- It is used to verify the selected RRM families from PFAM. RRMs have a very conserved fold and instead of blindly using all the PFAM families listed under the RRM clan, we checked that the structures within were really RRMs with the correct fold.
- To check that in the binding mode cluster definition, the pair of entries with the lowest similarity score were still similar in the orientation of the RNA with respect to the RRM. Aside from the visual inspection the RNAs were also aligned to each other (Figure S2) and the RMSD was computed for further evidence.
- In two different parts of the text the term “visual inspections” was an over-simplification of the actual procedure, in both cases referring to the alignment quality checks. Those parts have been rephrased accordingly in pages 8 and 9.

3. The model appears to be rather restrictive and works only for cluster 0 and no binding mode change can occur.

Indeed, we focus on our ‘cluster 0’, which captures what has been broadly described as the canonical RRM-RNA binding mode (2). We think that a better understanding of this binding mode is still needed and relevant, and despite this limitation, RRMs that fall in this binding mode can still bind a wide range of RNA sequences. We would have liked to study other clusters but the data scarcity did not allow us to develop an accurate method.

4. The negative test should be strengthened and should include other entries if possible.

This issue has also been similarly raised by reviewer #1 in comment #7. We have included a new validation section using all the RNAcompete data available for RRMs, consisting of 171 different proteins obtained from the CISBP-RNA Database. Using this data, we reconstruct RNA sequences that show very little preference for the RRM under study, which can be considered as negatives, and show that RRMScorer is able to distinguish between better and worse binders.

Other issues:

1. p.4. "the number of unique positions between both nucleotides" It is not clear if the authors meant positions only in A, only in B, or both?

It means the total number of different positions bound by A and B together. This is now clarified in the manuscript (page 5).

2. Will the results sensitive to the specific threshold of 5A?

The 5 angstrom threshold is a broadly used threshold for atomic interactions (3). Considering that we are using residue-nucleotide level connections, and are not trying to predict atom interactions, which could be more dependent on such a threshold, we do not expect significant changes in our scoring if this threshold changes. It is in this respect also important to not make the threshold too large, as this would start to pick up interactions with nucleotides neighbouring the closest (binding) one, and would so dilute the data.

Minor issues

1. Figures seems to be jumping around in order, and it makes it difficult to go back and forth.

Figure 1 and 8 have 2 and 3 different subparts, respectively, that we indeed refer to in different parts of the text. We have changed Figure 8 and references to it accordingly, but have left Figure 1 as is, because we think it is useful to have a representative RRM-RNA complex (figure 1A) next to the RRM schematic representation (figure 1B), even if we refer to Figure 1B later in the text.

List of the main changes made on the manuscript (all reviewers):

- New discussion on the knowledge-based potentials in the introduction, comparing them with our method (page 2)
- Introduction and discussion of RoseTTAFoldNA and how it could be coupled with RRMScorer (Introduction, page 2; Discussion, page 13)
- Addition of a missing dataset with the RRM-RNA complexes aligned (Dataset S6) and renaming of the following datasets (Datasets S7 to S10)
- New discussion on the cutoff effect for the RNA binding mode clustering. A new figure has been added on supplementary materials (Figure S2) and discussed to justify the cutoffs selection.
- Much deeper discussion on the RRMScorer equation, how it is adapted from the information difference equation and its relationship with the GOR method (page 6). The development from the information difference equation to the final RRMScorer equation has also been added as supplementary materials (equations S1 to S4).
- The error in the subscript of the RRMScorer equation has been fixed.
- The negative set has been appropriately renamed to randomized set.
- A new validation set has been added using RNAcompete data, the data processing is mentioned in the materials and methods section (page 7), and then the results are presented and discussed in the results and discussion sections respectively (page 11 and page 13).

References:

1. Garnier,J., Gibrat,J.F. and Robson,B. (1996) GOR method for predicting protein secondary structure from amino acid sequence. *Methods Enzymol*, **266**, 540–553.

2. Cléry,A., Blatter,M. and Allain,F.H.-T. (2008) RNA recognition motifs: boring? Not quite. *Current Opinion in Structural Biology*, **18**, 290–298.

3. Corley,M., Burns,M.C. and Yeo,G.W. (2020) How RNA-Binding Proteins Interact with RNA: Molecules and Mechanisms. *Mol Cell*, **78**, 9–29.
